# Effects of Plyometric Training on Sprint Running Performance in Boys Aged 9–12 Years

**DOI:** 10.3390/sports7100219

**Published:** 2019-10-10

**Authors:** Nobuaki Tottori, Satoshi Fujita

**Affiliations:** 1 Graduate School of Sport and Health Science, Ritsumeikan University, Kusatsu 525-8577, Japan; sh0037xp@ed.ritsumei.ac.jp; 2 Faculty of Sport and Health Science, Ritsumeikan University, Kusatsu 525-8577, Japan

**Keywords:** sprint performance, peak height velocity, youth sports, maximal sprint velocity, step frequency, step length

## Abstract

Skilled sprinting is fundamental in many sports, especially to improve athletic performance in youth. This study therefore aimed to investigate the effect of plyometric training on sprint performance in boys aged 9–12 years. Twenty boys were divided into a plyometric training group (*n* = 9) and a control training group (*n* = 11). In both groups, participants performed respective training programs once per week for 8 weeks with measurements at baseline and post-intervention. Sprint performance was assessed by 50-m sprint time, sprint velocity, step frequency and step length at 10-m intervals. Jumping performance was assessed using horizontal, vertical and rebound jumps. The plyometric training group showed an improved sprint velocity at 20–30 m, 30–40 m and 40–50 m, and step length at 0–10 m, 20–30 m and 30–40 m (*p* < 0.05). Furthermore, only the plyometric group showed an increased standing long jump distance and rebound jump performance (*p* < 0.05). The control group did not show any significant changes in any variable. Our findings suggest that plyometric training in pre-adolescent boys improves sprint velocity and step length at the maximum velocity phase concomitant with increased horizontal and rebound jump performance.

## 1. Introduction

High-level sprint performance is a necessary fundamental skill in many sports, in not only adults, but also in youth. Therefore, there has been high interest in improving fundamental skills, as successful athletes and the physical fitness of general children require the acquisition of a higher sprint performance at a younger age [1,2]. Many considerations are required to establish effective and efficient training methods for improving sprint performance, because youth athletes must attain high-level performance in many physical aspects of their sport (e.g., agility, power and sport-specific techniques).

As children mature, increases in body height, body mass, lower limb strength and neuromuscular function allow for concurrent improvements in sprint performance. Sprint velocity, which is a major variable in sprint performance, is calculated as the product of step frequency (SF) and step length (SL), also known as the spatiotemporal variables. It is necessary to increase one factor as long as the other factor is maintained or increased, since the relationship between the two factors is negatively correlated at maximum effort [3]. Along with other aspects of physical maturation, previous studies found that development of sprint velocity is associated with an increase in SL [4,5]. Although SL increases do continue into adulthood, there is a plateau of development in sprint performance associated with a decreased SF during 1.5–2.5 years before peak height velocity (PHV) in boys [4,5,6]. 

Nagahara et al. [5] reported that there is a stage of temporal slower development of sprint performance with a decrease in SF in Japanese boys aged 8.8–12.1 years. Therefore, research is needed to investigate the training methods to maintain or improve this SF in young boys aged before achieving PHV. However, training adaptations among spatiotemporal variables by any physical training have not been clarified in children.

Plyometric training is performed by exerting maximal muscular power in a short duration, and includes activities such as jumping, hopping, skipping and bounding. This training is a popular exercise for improving the performance of various athletic activities, including those using sprinting [7,8,9,10,11,12]. One meta-analysis revealed a moderate effect of plyometric training on sprint performance in healthy adults [11]. Moreover, sprint time was also significantly reduced after plyometric training in youth players who belong to a sports club, including soccer and tennis [7,8,12] and nonathletic children aged before achieving PHV [9,10]. Lloyd et al. [10] reported that a 6-week plyometric training program reduced the 10-m sprint time in non-athletic boys aged 1.5 ± 0.4 years before achieving PHV (age: 12.7 ± 0.7 years). Those findings suggested that plyometric training reduces sprint time regardless of whether it was performed in athletic or nonathletic children aged before achieving PHV. However, as we mentioned, adaptive mechanisms leading to the decrease in sprint time (e.g., increase in SF or SL) after plyometric training have not been clarified in children, although the definitive positive efficacy of this training on jumping and sprinting performance is evident. In male adult sprinters, two weeks of plyometric training decreased a 20-m sprint time through an increased SF, which was the result of a reduction in ground contact time during the support phase [13]. In contrast, Lockie et al. [14] reported that plyometric training decreased the 10-m sprint time through an increase in SL. To the best of our knowledge, no study has examined the mechanisms by which plyometric training reduces sprint time by changes in sprint spatiotemporal variables in children.

Thus, we aimed to investigate the effect of plyometric training on sprint performance including spatiotemporal variables in boys aged before achieving PHV. We hypothesized that plyometric training has the positive effect on sprint velocity derived from enhancements in both SF and SL in this age group.

## 2. Materials and Methods

### 2.1. Participants

Twenty Non-athletic boys aged 9–12 years from five public elementary schools participated in this study and were randomly assigned into two groups as follows: A plyometric training group (PG; *n* = 9) and a control-training group (CG; *n* = 11). All participants had no experience of plyometric training, and had never belonged to a track and field club. Their years from achieving their peak height velocity (PHV) was estimated by a regression equation from a previous study [15]. All participants were younger than the age of achieving PHV (Table 1). Their parents were informed of experimental procedures and provided written consent for their children to participate in the study. Additionally, the children’s assents were obtained. All procedures were approved by the Ethics Committee of Ritsumeikan University (BKC-IRB-2015-019).

The participant’s body height was measured to the nearest 0.1 cm using a stadiometer (Seca 213, Seca; Hamburg, Germany). Body mass was measured to the nearest 0.1 kg with the participant wearing light clothes using a bioelectrical impedance analysis device (Inbody720, Biospace, Cerritos, CA, USA). Body mass index (kg/m^2^) was calculated as the quotient of body mass divided by body height squared. Anthropometric characteristics of all participants are presented in Table 1.

### 2.2. Training Program

Both groups performed respective training programs in a gymnasium of the university once per week for eight weeks. The training duration was 60 min including warm-up, cool-down and adequate recovery between sets to minimize any risk of injury. In a previous study with same age children, Lloyd et al. [10] selected multiple sets of four exercises for plyometric training to allow sufficient repetition for developing motor control. The plyometric training program comprised of activities utilizing various types of vertical (vertical jumping, jump over barrier and tuck jump) and horizontal (long jump, power skip and bounding) jumps, according to previous studies [7,9,10,13,14]. Specifically, sprint-specific training (i.e., bounding) has been shown to have a positive effect upon sprint velocity [16]. A progressive overload of jumping was applied by varying the complexity of movement and gradually increasing the number of ground contacts up to 120 by the end of the training period (Table 2). The plyometric training group was required to perform the exercises with maximal effort (minimizing contact time and maximal jump height). CG performed recreational activities involving <10 m of running, such as playing catch with a ball and a cognitive-effort game for the purpose of creating no significant changes in the variables of this study. The length of a training session was approximately 60 min for both training groups.

### 2.3. Sprint Performance

Participants performed a maximal effort over a 50-m sprint twice from a standing start on an all-weather track following an adequate warm-up. A finish line was established at 55-m to run through the 50-m line. Between trials, participants had at least a 10-min rest. Participants were signaled to start the sprint by the sound of an electronic starting device (JESTER II, NISHI; Tokyo, Japan) and recorded by a computer as a trigger signal. The 50-m sprint running time (defined as the duration from the start signal to reaching the 50-m) was measured using photocells (E3G-R13, Omron; Kyoto, Japan). Sprint running motions through the entire 55-m sprint were recorded using a panning high-speed camera (GC-PX1, JVC; Kanagawa, Japan) collecting at 300 Hz. We used a video-analysis method for calculating spatiotemporal variables according to a previous study [17]. These cameras were placed at least 20 m from the right side of running lane at 25 m from the start line, and synchronized with the electronic starting device. Reference markers at 10-m intervals from the start line were placed 5 m beyond the running lane from each intersecting point between the extension lines of the panning camera, and every 10-m interval from the center of running lane and left side of the running lane.

For the image analysis process, the step characteristics were analyzed from the electronic flash by the starting device to the first step over the 50-m mark. The observer was asked to select the frame in which the pelvis was aligned with each of the five markers at 10-m intervals (F_justXm_), and the frame of the foot contact just before (F_beforeXm_) and after (F_afterXm_) each of the F_just_. Each 10-m interval time was calculated as the difference between F_justXm_ and F_justX-10m_, and divided by the frame rate (i.e., 300 Hz). The number of steps from starting to each F_beforeXm_ (N_0-Xm_) were counted by viewing the movies; subsequently, the number of steps at 10-m intervals (Step_X-X+10m_) were calculated by the Equation (1) for 0 m to 10 m and Equation (2), which shows an example of Step_10-20m_ for the others according to a previous study [18]. Step frequency (SF) was calculated as the quotient of Step_X-X+10m_ and each 10-m interval time. The step length (SL) was calculated as ten divided by the each Step_X-X+10m_. The running velocity was calculated as the product of SL and SF. Additionally, each maximal value and the average of the 10-m interval value were used for post analysis.

Step_0-10m_ = N_0-10m_ + (F_just10m_ − F_before10m_/F_after10m_ − F_before10m_) (1)

Step_10-20m_ = N_0-20m_ − Step_0-10m_ + (F_just20m_ − F_before20m_/F_after20m_ − F_before20m_) (2)

### 2.4. Jumping Performance

Three types of vertical jumps were performed on a force platform (TF6090, Tec Gihan Co., Ltd., Kyoto, Japan; 1000 Hz) as follows: Countermovement jump (CMJ), squat jump (SJ), and rebound jumps (RJ). Prior to the performance test, participants performed a specific warm-up (i.e., three sub maximal jumps each). For CMJ, participants started from an upright standing position and completed a rapid leg extension immediately after a fast-downward movement by flexing the hips and knees. For SJ, participants started from a stationary semi-squat position (knee angle of 90°) and performed the jump with maximal effort. For RJ, participants started from an upright position and performed six consecutive jumps in which they were instructed to jump as high as possible and push against the ground as quickly as possible. Participants performed all vertical jumps with their hands on their hips and their elbows pointing away from their body in order not to swing their arms. The trials were completed three times with a 30-s recovery period between them, according to a previous study [19]. The vertical ground reaction forces of each single jump were recorded using an AD converter (Power Lab 16/30, ADInstruments Pty Ltd.; Sydney, Australia) and software (LabChart ver. 7, ADInstruments Pty Ltd.; Sydney, Australia) connected to the force platform. The takeoff phase was defined as the point in which the force first became zero after standing on the platform. The landing phase was defined as the point at which the force started to increase. Flight-time was the duration from the take-off phase to the landing phase. Jump height was determined using an established flight-time calculation method [20]. Only height was measured during CMJ and SJ, and height, contact time (RJCT) and jump index (RJ-index; calculated as the ratios of height divided by the RJCT) were measured during RJ [21]. The RJ-index is used to assess the capacity to rapidly exert force under a high eccentric load similar to the reactive strength index [22]. The highest values of jump height or RJ-index trials were used in the post analysis.

The standing long jump (SLJ) was used as a measure of horizontal lower body muscular strength [23]. Participants jumped from a line in the front horizontal direction with both legs using an arm swing. Following two submaximal practice jumps, participants performed two trials. The distance was measured from the line to the nearest contact of any body part with the ground. The longest distance of all trials was used for further analysis. The reliability of the jumping measurement, which was determined by the intra class correlation coefficient (ICC), were 0.970 for SJ, 0.962 for CMJ, 0.944 for RJ-index and 0.953 for the standing long jump (SLJ), respectively.

### 2.5. Statistical Analysis

Data are expressed as mean ± standard deviation (mean ± SD) with 95% confidence intervals (95% CI). An independent sample t-test was used to compare anthropometric variables between groups at baseline. The effects of training were analyzed for statistical significance using a repeated measurement two-way Analysis of Variance (ANOVA) (time [pre, post] × group [PG, CG]). When significant group-by-time interactions were observed, group-specific post hoc tests (i.e., paired t-test) were used to identify statistically significant differences. Additionally, we calculated the mean difference (Δ) and 95% CI of changes in variables. Effect sizes were calculated using partial eta square for ANOVA and Cohen’s d for post hoc tests. Cohen’s d was classified as small (0.20 ≤ d ≤ 0.50), medium (0.50 ≤ d ≤ 0.80), and large (0.80 ≤ d) [24]. The alpha level was set at *p* < 0.05 to indicate statistical significance. All statistical analyses were performed using the SPSS statistical software version 25 (SPSS Inc.; Chicago, IL, USA).

## 3. Results

There were no significant differences in variables among demographic, anthropometric, sprint performance and jumping performance between groups at the baseline. The CG did not show any significant changes in measurement variables after intervention. Significant group-by-time interactions were observed for 50-m sprint time (*F* = 12.673, *p* = 0.002, η_p_^2^ = 0.413), maximal velocity (*F* = 7.797, *p* = 0.012, η_p_^2^ = 0.302) and 50-m SL (*F* = 5.401, *p* = 0.032, η_p_^2^ = 0.231) (Table 3). In the PG, post hoc analysis revealed a significant decrease in 50-m sprint time (*p* = 0.005, d = −0.442) and increase in maximal velocity (*p* = 0.031, d = 0.489). An increase in 50-m SL tended to be significant (*p* = 0.064, d = 0.235).

Regarding 10-m interval characteristics, Table 4, Table 5 and Table 6 show the changes in sprint velocity, SL and SF, respectively. Significant group-by-time interactions were observed for sprint velocity for 10–20 m, 20–30 m, 30–40 m, and 40–50 m intervals (*F* = 4.548–10.005, *p* < 0.05 for all, η_p_^2^ = 0.202–0.357). In PG, post hoc analysis showed significant increases in sprint velocity during 20–30 m (*p* = 0.033, d = 0.424), 30–40 m (*p* = 0.002, d = 0.474) and 40–50 m intervals (*p* = 0.026, d = 0.398). In addition, a significant interaction for SL for 0–10 m and 20–30 m intervals was observed (*F* = 8.620 and 5.820, *p* = 0.009 and 0.027, η_p_^2^ = 0.324 and 0.244, respectively). In PG, post hoc analysis showed a significant increase in SL during the 0–10 m (*p* < 0.001, d = 0.258), 20–30 m (*p* = 0.037, d = 0.281) and 30–40 m intervals (*p* = 0.038, d = 0.389). There were no significant interactions observed for SF.

Statistical analysis showed significant interactions for SLJ (*F* = 4.530, *p* = 0.047, η_p_^2^ = 0.201) and the RJ-index (*F* = 8.189, *p* = 0.010, η_p_^2^ = 0.313). An interaction for RJCT tended to be significant (*F* = 3.682, *p* = 0.071, η_p_^2^ = 0.170) (Table 7). The in PG, post hoc analysis revealed significant increases in SLJ (*p* = 0.005, d = 0.364) and the RJ-index (*p* = 0.019, d = 0.542), and a decrease in RJCT (*p* = 0.026, d = −0.571). There were no main effects and interactions in SJ, CMJ and RJ height.

## 4. Discussion

The aim of the present study was to investigate the effects of plyometric training on sprint performance including SF and SL in boys aged 9–12 years. Our study results showed that plyometric training has positive effects on sprint velocity and SL during the maximum velocity phase in young boys aged before achieving their PHV. In addition, small to medium increases in RJ performance and SLJ were observed. To our knowledge, this is the first study to clarify that the adaptive mechanism of improvement in sprint time was driven by an increase in SL in pre-adolescent boys.

Our primary finding was the significant positive effect of plyometric training on SL, not SF. This result was in line with the previous findings conducted by Lockie et al. [14], which showed that 6-week plyometric training improved SL in male field sports players. The reduction of ground contact time is related to an increase in SF during sprint running [3]. Although the program of this present study significantly reduced RJCT, SF did not increase. The present study also revealed an increasing trend in horizontal lower muscular strength (i.e., distance in SLJ). A previous study reported that an increase in horizonal power is related to the increase in step length [14]. Our study also found a significant improvement of step length in 0–10 m. In addition, the distance during SLJ is related to flight time; horizontal jumps are negatively related to SF [25]. Therefore, the present training program may maintain SF. In contrast, Mackala et al. [13] showed that two weeks of plyometric training, including various types of jumping, significantly increased vertical and horizontal jumping. Furthermore, the increase in vertical jumps was greater than in horizonal jump in male sprinters [13]. That study also reported that the program increased only SF, but not SL [13]. Aforementioned, there is a slower temporal development of sprint performance with a decrease in SF in boys aged before achieving PHV [5]. Therefore, our findings suggest that plyometric training might maintain SF in boys aged before achieving PHV. Future studies are needed to examine the effects on spatiotemporal variables in children of different age groups and the relationships between various types of training, jumping performance and spatiotemporal variables.

There were significant improvements of sprint velocity during 20–30 m, 30–40 m and 40–50 m intervals, which are categorized as the secondary acceleration and maximal velocity phases. This supports a previous study that reported that sprint time during the 10–20 m and 20–30 m, but not the 0–10 m intervals, were improved after plyometric training [9]. In addition, Diallo et al. [26] and Sohnlein et al. [12] showed a greater decrease in 20-m sprint time after plyometric training than in that of the control group without exercise intervention in prepubescent soccer players; however, changes in 5-m and 10-m sprint time were not observed. The plyometric training program performed in the present study mainly consisted of repeated jumps requiring the shortest ground contact time possible (i.e., jumping over barriers, tuck jumps, power skips and bounding). Therefore, the RJ-index significantly increased after training intervention. Bret et al. [27] reported that the hopping test, like the RJ test, is related to sprint velocity after 30 m, and the height of the CMJ is related to sprint velocity before 30 m in national level male sprinters. Therefore, in the present study, the sprint velocity during the maximal velocity phase was improved as reflected by an increase in the RJ-index. However, another previous study in male field sport athletes reported that plyometric training decreases 5-m sprint time, but not 10-m sprint time [14]. These contrary results could be explained by differences in plyometric training modes and participant characteristics. In the present study, SJ and CMJ, which are related to sprint velocity during the acceleration phase by previous studies [21,27], did not exhibit improvements. We suggest that changes in sprint velocity during the initial acceleration phase were not observed in the present study owing to the lack of changes in SJ and CMJ performance.

Our study results have practical relevance for the methods of plyometric training used in pre-adolescent boys. Strength and conditioning coaches and physical education teachers should note that plyometric training, such as that used in this study, improves RJ performance and SL, particularly during the maximal running phase. In addition, if coaches wish to improve SF, other training modalities (e.g., assisted sprint training, as described by Macadam et al. [28]) should be added to the present program. However, the optimal method to improve SF in pre-adolescent children needs to be clarified.

Furthermore, the present study observed a reduced 50-m sprint time as performed once per week for eight weeks (−2.78%, d = −0.442). A previous study showed significant decreases in 20-m sprint time (−2.94%, d = −0.45) after twice-weekly plyometric training for six weeks in prepubertal children [10]. According to these results, increased training volume and frequency may not afford a larger effect of training. In adults, low-to-moderate training frequency (once per week or twice per week) produces a similar effect on jumping and sprint performance as that with higher training frequency (three times per week) [29]. Regarding training volume within a single session, Chaabene et al. [7] reported similar improvements on sprint time and jump performance between low (60–120 jumps per session) and high (110–220 jumps per session) volume training in pre-adolescent children. A meta-analysis that summarized the effects of plyometric training on jumping and sprint performance in healthy adults revealed no significant association between the total changes in sprint performance and the total number of ground contacts [11]. Those findings suggest that plyometric training has equally efficacious methods to improve sprint performance, even with a lower training volume. The program used in the present study may be effective and time-efficient for improving sprint performance in both active and nonactive children.

The present study has some limitations. First, we did not assess ground contact and aerial times during sprint running. Second, we measured the mean values of spatiotemporal variables at 10-m intervals; thus, changes in variables for each step were not determined. Recently, there are different strategies of ascertaining changes in spatiotemporal variables for each step, and these variables are affected by age [5] and athletic level [30]. To the best of our knowledge, no study has revealed the effect of training on spatiotemporal variables and kinetic values for each step during 50-m or 100-m events. Thus, future studies are needed to determine the effect of training on variables during each step. Third, either the exercise intensity or volume in CG was not matched with PG. It is difficult to match the exercise intensity or volume of other exercise modalities with the plyometric training because the training volume of plyometric training depends only on the number of ground contacts, and the exercise intensity of plyometric training depends on the height of the jumps. Accordingly, exercise volume or intensity on plyometric training cannot be directly translated into other exercise modality with difference movements. However, the length of a training session was equated for approximately 60 min for both training groups.

## 5. Conclusions

To our knowledge, this is first study to reveal the positive effects of plyometric training on sprint velocity and SL during secondary acceleration and the maximal velocity phase in nonathletic pre-adolescent boys. In addition, the plyometric training program performed in this study significantly improved RJ and SLJ performance, but not SJ and CMJ, wherein previous studies reported a positive effect. Future studies are needed to clarify the methods of improving SF in boys aged before achieving PHV. Strength and conditioning coaches and physical education teachers should note that plyometric training, such as that used in this study, improves RJ performance and SL, particularly at the maximal running phase.

## Figures and Tables

**Table 1:** Participants’ baseline demographics.

Variables	Control Training Group			Plyometric Training Group		
n	11			9		
Age (yrs.)	10.9	±	0.9	10.3	±	0.7
Y-PHV	−3.1	±	0.5	−3.5	±	0.6
Body height (cm)	142.3	±	6.8	138.6	±	7.9
Body mass (kg)	35.2	±	6.4	32.1	±	5.6
BMI (kg/m^2^)	17.3	±	2.4	16.6	±	1.7

Values are mean ± SD; Y-PHV indicates the year from achieving the peak height velocity.

**Table 2:** Number of ground contacts in each plyometric training session.

Exercises	Week							
	1	2	3	4	5	6	7	8
Vertical jump	10	10	10	10	10	10	10	10
Jump over barrier	20	20	30	30	20	20	20	20
Tuck jump				5	10	20	20	30
Long jump	10	10	10	10	10	10	10	10
Power skip	40	40	50	50				
Bounding				10	40	40	50	50
Total	80	80	100	115	90	100	110	120

**Table 3:** Changes in 50-m sprint performance after training intervention.

50-m Sprint	Control Training Group			Plyometric Training Group			ANOVA *p*		
	Pre	Post	Δ (95% CI)	Pre	Post	Δ (95% CI)	Time	Group	Group × Time
Sprint time (s)	9.41 ± 0.90	9.54 ± 1.04	0.13 (−0.06–0.32)	9.71 ± 0.65	9.44 ± 0.58 *	−0.27 (−0.44–−0.11)	0.227	0.778.	**0.002**
Mean SF (step/s)	3.86 ± 0.27	3.88 ± 0.31	0.02 (−0.08–0.12)	3.69 ± 0.22	3.73 ± 0.24	0.04 (−0.03–0.12)	0.251	0.182	0.711
Mean SL (m/step)	1.39 ± 0.09	1.36 ± 0.08	−0.03 (−0.06–0.01)	1.40 ± 0.10	1.43 ± 0.09	0.02 (0.00–0.05)	0.877	0.315	**0.032**
MAX V (m/s)	6.11 ± 0.54	6.01 ± 0.63	−0.10 (−0.26–0.05)	5.79 ± 0.36	5.96 ± 0.32 *	0.17 (0.02–0.31)	0.523	0.394	**0.012**
MAX SF (step/s)	4.18 ± 0.32	4.20 ± 0.36	0.02 (−0.12–0.16)	3.92 ± 0.17	3.96 ± 0.22	0.04 (−0.02–0.09)	0.447	0.515	0.838
MAX SL (m/step)	1.51 ± 0.11	1.51 ± 0.11	0.00 (−0.06–0.06)	1.53 ± 0.12	1.55 ± 0.11	0.02 (−0.02–0.07)	0.554	0.056	0.498

Note: Values are mean ± SD; 95% CI, 95% confidence interval; SF, step frequency; SL, step length; Bold text indicates statistical significance (*p* < 0.05); * *p* < 0.05, significant difference within group. ANOVA refers to Analysis of Variance.

**Table 4:** Changes in sprint velocity after training intervention.

Sprint Velocity (m/s)	Control Training Group			Plyometric Training Group			ANOVA *p*		
	Pre	Post	Δ (95% CI)	Pre	Post	Δ (95% CI)	Time	Group	Group × Time
0–10 m	3.79 ± 0.30	3.81 ± 0.29	0.02 (−0.02–0.05)	3.82 ± 0.41	3.93 ± 0.33	0.12 (−0.05–0.28)	0.063	0.613	0.148
10–20 m	5.97 ± 0.45	5.86 ± 0.57	−0.11 (−0.28–0.05)	5.68 ± 0.34	5.81 ± 0.33	0.13 (−0.07–0.33)	0.885	0.390	**0.047**
20–30 m	6.09 ± 0.60	5.99 ± 0.64	−0.10 (−0.21–0.02)	5.76 ± 0.35	5.91 ± 0.35 *	0.15 (0.02–0.28)	0.503	0.393	**0.005**
30–40 m	5.97 ± 0.64	5.88 ± 0.62	−0.09 (−0.24–0.07)	5.68 ± 0.40	5.86 ± 0.34 *	0.17 (0.08–0.27)	0.324	0.519	**0.006**
40–50 m	5.88 ± 0.61	5.75 ± 0.60	−0.13 (−0.34–0.07)	5.59 ± 0.35	5.74 ± 0.40 *	0.15 (0.02–0.28)	0.901	0.517	**0.023**

Note: Values are mean ± SD; 95% CI, 95% confidence interval; Bold text indicates statistical significance (*p* < 0.05); * *p* < 0.05, significant difference within group.

**Table 5:** Changes in step length after training intervention.

Step Length (m)	Control Training Group			Plyometric Training Group			ANOVA *p*		
	Pre	Post	Δ (95% CI)	Pre	Post	Δ (95% CI)	Time	Group	Group × Time
0–10 m	1.12 ± 0.06	1.10 ± 0.05	−0.02 (−0.05–0.01)	1.13 ± 0.08	1.15 ± 0.08 *	0.02 (0.02–0.03)	0.908	0.290	**0.009**
10–20 m	1.44 ± 0.11	1.41 ± 0.10	−0.04 (−0.08–0.01)	1.46 ± 0.10	1.47 ± 0.10	0.02 (−0.04–0.07)	0.576	0.352	0.092
20–30 m	1.49 ± 0.11	1.47 ± 0.09	−0.02 (−0.06–0.02)	1.50 ± 0.11	1.52 ± 0.10 *	0.03 (0.00–0.06)	0.683	0.479	**0.027**
30–40 m	1.49 ± 0.11	1.47 ± 0.11	−0.02 (−0.08–0.05)	1.50 ± 0.11	1.54 ± 0.11 *	0.04 (0.00–0.08)	0.511	0.417	0.121
40–50 m	1.49 ± 0.11	1.45 ± 0.11	−0.04 (−0.10–0.03)	1.53 ± 0.12	1.53 ± 0.12	0.00 (−0.04–0.04)	0.404	0.248	0.324

Note: Values are mean ± SD; 95% CI, 95% confidence interval; Bold text indicates statistical significance (*p* < 0.05); * *p* < 0.05, significant difference within group.

**Table 6:** Changes in sprint frequency after training intervention.

Step Frequency (Hz)	Control Training Group			Plyometric Training Group			ANOVA *p*		
	Pre	Post	Δ (95% CI)	Pre	Post	Δ (95% CI)	Time	Group	Group × Time
0–10 m	3.38 ± 0.23	3.45 ± 0.26	0.07 (0.00–0.15)	3.37 ± 0.36	3.42 ± 0.32	0.04 (−0.11–0.19)	0.112	0.864	0.672
10–20 m	4.15 ± 0.32	4.17 ± 0.37	0.02 (−0.13–0.17)	3.91 ± 0.16	3.95 ± 0.22	0.04 (−0.03–0.11)	0.470	0.074	0.763
20–30 m	4.09 ± 0.33	4.08 ± 0.36	−0.01 (−0.11–0.09)	3.86 ± 0.19	3.89 ± 0.23	0.03 (−0.04–0.10)	0.837	0.114	0.510
30–40 m	4.01 ± 0.31	4.00 ± 0.40	−0.01 (−0.19–0.17)	3.80 ± 0.19	3.82 ± 0.24	0.01 (−0.08–0.11)	0.924	0.145	0.840
40–50 m	3.95 ± 0.33	3.96 ± 0.36	0.00 (−0.16–0.16)	3.68 ± 0.25	3.76 ± 0.23	0.09 (−0.02–0.19)	0.330	0.084	0.371

Note: Values are mean ± SD; 95% CI, 95% confidence interval.

**Table 7:** Changes in jump performance after training intervention.

Jump	Control Training Group			Plyometric Training Group			ANOVA *p*		
	Pre	Post	Δ (95% CI)	Pre	Post	Δ (95% CI)	Time	Group	Group × Time
SLJ (cm)	163.8 ± 19.7	164.95 ± 20.28	1.14 (−2.82–5.09)	159.94 ± 17.32	166.41 ± 18.25 *	6.47 (2.51–10.43)	**0.007**	0.888	**0.047**
CMJ (cm)	23.46 ± 3.96	23.16 ± 4.83	−0.30 (−1.81 –1.20)	21.52 ± 3.34	22.77 ± 3.56	1.25 (−0.57–3.08)	0.369	0.510	0.150
SJ (cm)	22.03 ± 3.31	21.96 ± 4.40	−0.06 (−1.72 –1.60)	21.16 ± 2.83	22.56 ± 3.22	1.40 (−0.37–3.16)	0.231	0.929	0.192
RJ-index (m/s)	0.82 ± 0.28	0.78 ± 0.25	−0.04 (−0.12 –0.04)	0.89 ± 0.18	1.00 ± 0.22 *	0.11 (0.02–0.19)	0.187	0.167	**0.010**
RJCT (s)	0.22 ± 0.03	0.23 ± 0.04	0.01 (−0.01–0.03)	0.20 ± 0.02	0.19 ± 0.02 *	−0.01 (−0.02–0.00)	0.929	**0.010**	0.071
RJ height (cm)	17.60 ± 4.78	17.42 ± 3.17	−0.18 (−2.51 –2.15)	17.71 ± 3.53	18.78 ± 3.98	1.07 (−0.58–2.72)	0.511	0.658	0.359

Note: Values are mean ± SD; 95% CI, 95% confidence interval; SLJ, standing long jump; CMJ, countermovement jump; SJ, squat jump; RJ-index, rebound jump index; RJCT, contact time of rebound jump; RJ height, rebound jump height; Bold text indicates statistical significance (*p* < 0.05); * *p* < 0.05, significant difference within group.
